# SIRT3 regulates PDHA1 acetylation in HUVECs to modulate inflammation and pyroptosis under clinorotation

**DOI:** 10.1016/j.isci.2025.113790

**Published:** 2025-10-16

**Authors:** Min Jiang, Junjie Shao, Kun Lin, Lejian Lin, Shuai Yue, Haojie Yan, Jingjing Zhou, Shujin Shi, Xin Li, Ran Zhang

**Affiliations:** 1 Department of Pulmonary and Critical Care Medicine, Chinese PLA General Hospital, Beijing 100853, China; 2 Department of Cardiovascular Medicine, Chinese PLA General Hospital, Beijing 100853, China; 3 Graduate School of Chinese PLA Medical School, Beijing 100853, China; 4 Outpatient Department, The First Medical Center of Chinese PLA General Hospital, Beijing 100853, China; 5 Division of Health Services, The First Medical Center of Chinese PLA General Hospital, Beijing 100853, China; 6 State Key Laboratory of Kidney Diseases & National Clinical Research Center for Kidney Diseases, Chinese PLA General Hospital, Beijing 100853, China

**Keywords:** cell biology, microgravity sciences

## Abstract

Microgravity-induced endothelial inflammation contributes to cardiovascular dysfunction in astronauts, but the metabolic mechanisms involved are not fully defined. Sirtuin-3 (SIRT3), a mitochondrial deacetylase, regulates cellular metabolism and redox balance. The results demonstrate that two-dimensional clinorotation-induced simulated microgravity suppresses SIRT3 in human umbilical vein endothelial cells (HUVECs), resulting in mitochondrial dysfunction, NLRP3 inflammasome activation, and pyroptosis. SIRT3 overexpression mitigated these effects, while SIRT3 knockdown exacerbated them. Mechanistically, SIRT3 deletion promoted acetylation of pyruvate dehydrogenase E1α (PDHA1) at lysine 83, inhibiting pyruvate dehydrogenase complex (PDHC) activity and shifting metabolism toward higher levels of glycolysis. PDHA1 transfection suppressed NLRP3 inflammasome activation, pyroptosis, and glycolysis in HUVECs under simulated microgravity, while restoring mitochondrial membrane potential (ΔΨm) and oxidative phosphorylation. The PDHA1-K83R mutant provided stronger protection than wild-type PDHA1. These findings reveal that the SIRT3-PDHA1 axis links mitochondrial metabolism to endothelial inflammation under simulated microgravity, suggesting that targeting this pathway could help maintain vascular health during spaceflight.

## Highlights

• Endothelial dysfunction, exacerbated by microgravity, increases cardiovascular risk
• Simulated microgravity downregulates SIRT3, activating NLRP3 inflammasome and pyroptosis
• SIRT3 inhibition promotes glycolysis over oxidative phosphorylation, driving inflammation
• SIRT3 deacetylates PDHA1 at K83 to modulate metabolism in ECs under simulated microgravity

## Graphical abstract

**Figure:**
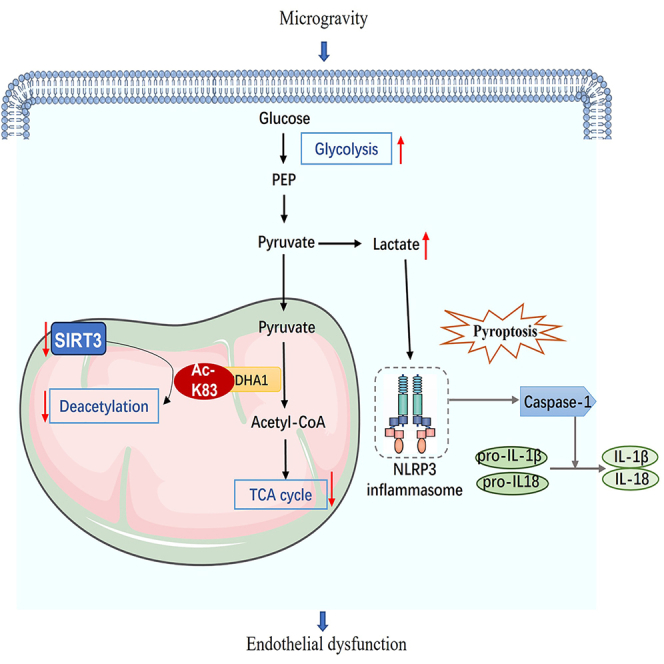

## Introduction

Spaceflight increases the risk of cardiovascular events.^1^ The effects of microgravity exposure on the human cardiovascular system are of great concern. The vascular endothelium, the most extensive secretory interface in the body, plays an important role in maintaining vascular homeostasis. Vascular endothelial cells (ECs) are highly sensitive to hypergravity and microgravity. In fact, they are susceptible to morphological and functional changes in response to changes in gravity. Microgravity alters the ability of ECs to grow in three dimensions,^2^ disrupts energy and protein metabolism,^3^ inhibits the expression of host defense-related genes,^4^ and regulates the expression of genes related to cell adhesion, oxidative phosphorylation (OXPHOS), and stress response.^5^ Endothelial dysfunction is associated with an increased risk of thrombosis^6^^,^^7^ and leads to post-flight immune dysfunction^8^ and atherosclerosis.^9^ However, the underlying mechanisms driving endothelial dysfunction under microgravity as well as the molecular mechanisms underlying EC responses to changes in gravity remain unknown.

The studies under outer-space microgravity and ground-based microgravity simulation confirm that inflammation leads to endothelial dysfunction. Under microgravity, the pro-inflammatory cytokines interleukin (IL)-1α and IL-1β were significantly increased in human umbilical vein endothelial cells (HUVECs),^10^^,^^11^ while the senescence-associated gene *CDKN1A* was upregulated and the anti-aging protein SIRT2 (sirtuin-2) was downregulated.^12^ We previously reported that vascular cell adhesion molecule-1 and E-selectin were highly expressed in the vascular endothelium.^13^^,^^14^^,^^15^ We also found that NLRP3 inflammasome was activated and the secretion of IL-6, IL-1β, IL-8, and tumor necrosis factor (TNF)-α was increased in HUVECs after 2D clinorotation.^16^ Activation of NLRP3 inflammasome leads to caspase-1-mediated secretion of IL-1β and IL-18 and pyroptosis, also referred to as inflammatory necrosis, which is associated with the rupture of cell membranes and the release of pro-inflammatory cytokines.^17^ NLRP3 inflammasome activation leads to senescence and endothelial dysfunction.^18^ However, the underlying mechanism of NLRP3 inflammasome activation in HUVECs under simulated microgravity remains unclear.

Numerous signals can activate NLRP3 inflammasome. Previous studies showed that blocked mitophagy leads to the accumulation of damaged, reactive oxygen species (ROS)-producing mitochondria, which in turn activates the NLRP3 inflammasome, whereas scavenging of mitochondria-specific ROS with mito-TEMPO inhibits activation of the NLRP3 inflammasome.^19^ Emerging evidence suggests that cellular metabolism plays a key role in the activation of NLRP3 inflammasome.^20^ Dysregulated protein metabolism and impaired host immunity have been found in space-flown HUVECs; also, metabolites associated with energy deprivation, including amino acids and cofactors such as lactate and pyruvate, have been identified.^3^ The augmented glycolytic capacity and activation of glycolysis-dependent mitochondrial respiration promote the activation of NLRP3 inflammasomes in macrophages.^21^ Glycolysis is a key pathway for cellular glucose metabolism, and its metabolite pyruvate is either branched to acetyl-CoA via pyruvate dehydrogenase complex (PDHC)-dependent-pyruvate decarboxylation or metabolized to lactate by lactate dehydrogenase A. PDHC not only connects glycolysis to OXPHOS but also influences pyruvate metabolism. Previously, a study has determined that glycolysis and pyruvate metabolism are affected by long-duration spaceflight in astronauts.^22^ As for ECs, Barravecchia et al. reported that genes related to OXPHOS were upregulated under microgravity,^23^ while Locatelli et al. showed that simulated microgravity inhibited oxygen consumption and maximal respiratory capacity.^24^ Therefore, further studies regarding the discrepancies and the underlying mechanism are needed.

SIRT3 (sirtuin-3) is a mitochondrial, NAD^+^-dependent deacetylase that regulates a variety of metabolic enzymes, including PDHA1, the first constitutive enzyme of PDHC, and inhibits the activation of the NLRP3 inflammasome.^25^ These findings suggest that SIRT3 may regulate the pro-inflammatory state through its influence on cellular metabolism. Therefore, the objective of this study was to investigate the potential involvement of SIRT3-regulated PDHA1 activity and glucose metabolism in the activation of the NLRP3 inflammasome and endothelial pyroptosis under simulated microgravity. Understanding these mechanisms will provide valuable insights into the pathogenesis of endothelial dysfunction and contribute to the development of effective measures for maintaining cardiovascular health.

## Results

### Effects of clinorotation on SIRT3 expression under simulated microgravity

**Figure 1:**
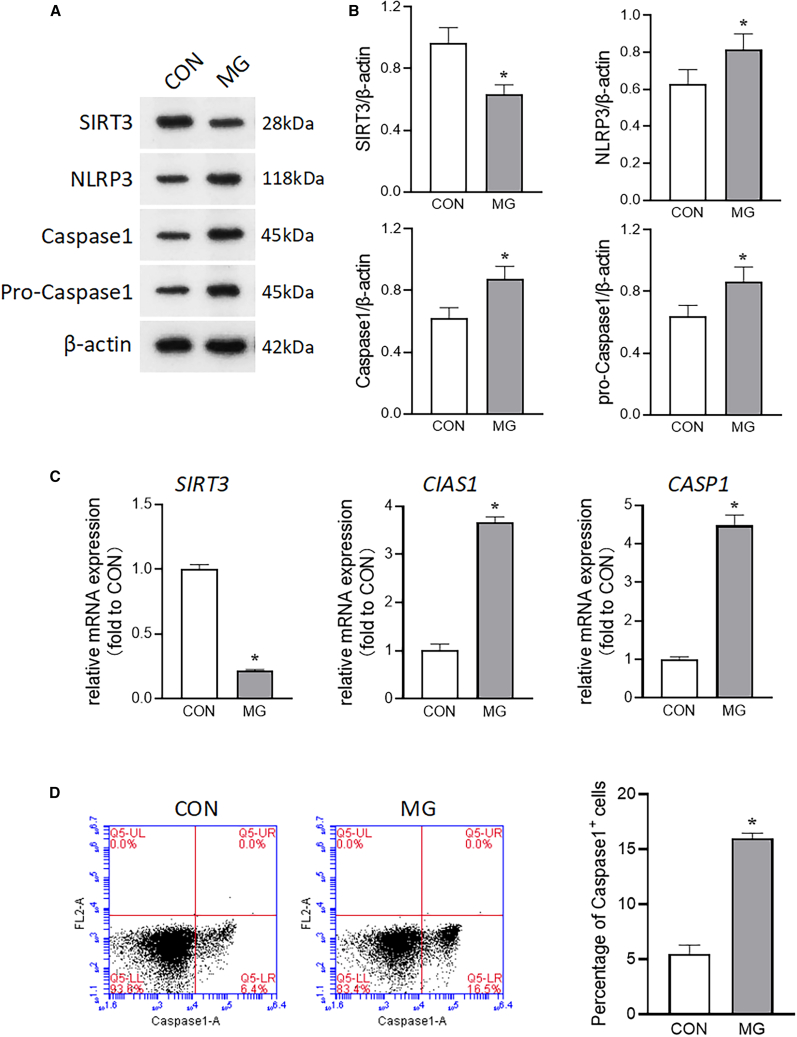
Expression of SIRT3 and NLRP3 inflammasome in microgravity-treated HUVECs (A) Representative blots displaying the expression of SIRT3, NLRP3, caspase-1, and pro-caspase-1 in HUVECs exposed to clinorotation for 72 h. (B) Semiquantitative densitometric analysis of the indicated protein levels. (C) Relative mRNA expression levels of *SIRT3*, *CIAS1*, and *CASP1* in HUVECs subjected to clinorotation for 72 h. (D) The proportion of caspase-1-positive cells in HUVECs subjected to clinorotation for 72 h mean ± SEM; *n* = 3 biological samples per group in triplicate were measured. Student’s t test: ∗*p* < 0.05 vs. CON.

We have previously reported the role of NLRP3 inflammasome activation in initiating endothelial dysfunction.^16^ To further investigate the role of SIRT3 in regulating inflammation under simulated microgravity, we examined the levels of SIRT3 and NLRP3 inflammasome-associated proteins. As shown in Figures 1A–1C, the mRNA and protein abundance of SIRT3 decreased under simulated microgravity, while the mRNA and protein levels of NLRP3 and caspase-1 were significantly increased. The formation of cleaved caspase-1 (Figure 1A) and the proportion of caspase-1-positive cells were significantly increased under simulated microgravity (Figure 1D), suggesting that the inhibition of SIRT3 under microgravity was associated with the activation of the NLRP3 inflammasome.

### Role of SIRT3 downregulation in pyroptosis and mitochondrial dysfunction under simulated microgravity

**Figure 2:**
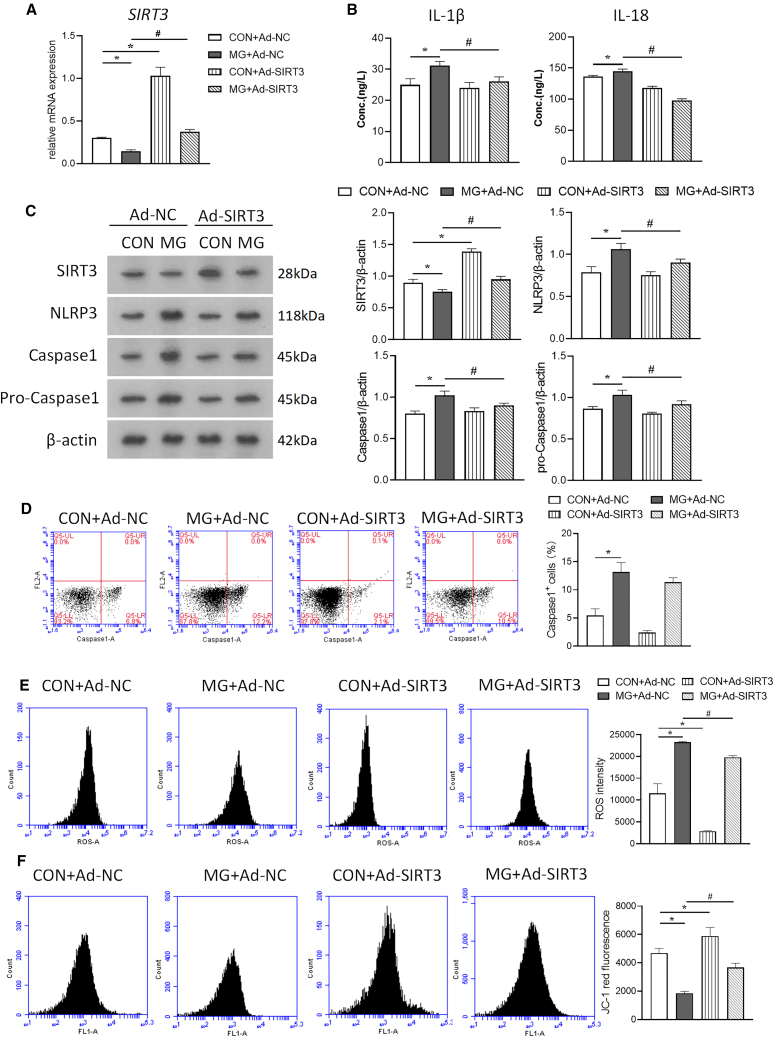
Effect of SIRT3 overexpression on NLRP3 inflammasome activation and pyroptosis in microgravity-treated HUVECs Adenovirus overexpressing SIRT3 (Ad-SIRT3) and its non-targeting sequences (Ad-NC) were transfected to HUVECs 48 h before clinorotation. (A) Relative mRNA expression level of *SIRT3* in HUVECs. (B) Concentration of IL-1β and IL-18. (C) The protein expression levels of SIRT3, NLRP3, caspase-1, and pro-caspase-1. (D) The proportion of caspase1-positive cells. (E) The cellular ROS level. (F) The JC-1 red fluorescence in HUVECs subjected to clinorotation for 72 h mean ± SEM; *n* = 3 biological samples per group in triplicate were measured. Tukey’s Honest Significant Difference (HSD) test: ***∗****p* < 0.05 vs. CON + Ad-NC, ^***#***^*p* < 0.05 vs. MG + Ad-NC.

**Figure 3:**
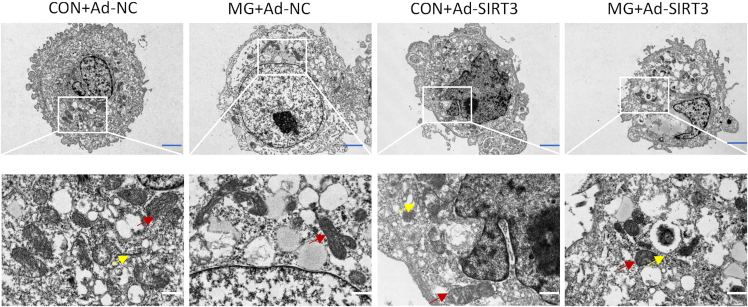
Effect of SIRT3 overexpression on cellular ultrastructure in microgravity-treated HUVECs Representative transmission electron microscopy images depicting the cellular ultrastructure (red arrows highlighting mitochondria, and yellow arrows indicating the endoplasmic reticulum). Blue scale bars, 2 μm; white scale bars, 500 nm.

To explore the role of SIRT3 in regulating pyroptosis in HUVECs under simulated microgravity, Ad-SIRT3 was transfected and the mRNA level of SIRT3 was examined (Figure 2A). As shown in Figure 2B, HUVECs overexpressing SIRT3 reduced the elevated levels of IL-1β and IL-18 in the MG group (Figure 2B). SIRT3 also reversed the increase in NLRP3, caspase-1, and pro-caspase-1 protein abundance under simulated microgravity (Figure 2C). The proportion of caspase-1-positive HUVECs was used to determine the pyroptosis rate. We found that the pyroptosis rate was elevated after clinorotation and SIRT3 overexpression inhibited pyroptosis (Figure 2D). Mitochondrial dysfunction may dissipate mitochondrial membrane potential (ΔΨm), leading to oxidative stress, which triggers activation of the NLRP3 inflammasome.^26^ Dihydroethidium (DHE) staining showed that clinorotation caused a significant increase in cellular ROS compared with control (Figure 2E). As shown in Figure 2F, the intensity of red fluorescence of JC-1 staining was significantly reduced in HUVECs after clinorotation compared with control, indicating a decrease in mitochondrial ΔΨm. In contrast, overexpression of SIRT3 significantly attenuated cellular ROS production and partially restored ΔΨm (Figures 2E and 2F). Electron microscopy revealed that HUVECs from the CON + Ad-NC and CON + Ad-SIRT3 groups displayed normal morphology; however, HUVECs in the MG + Ad-NC group exhibited morphological features of pyroptosis, such as membrane collapse, cytoplasmic swelling, cytoplasmic vacuolization, and swelling of the endoplasmic reticulum and mitochondria. Transfection of SIRT3 partially attenuated cellular ultrastructural damage under simulated microgravity (Figure 3). These data suggest that mitochondrial dysfunction is involved in pyroptosis of HUVECs under simulated microgravity and that SIRT3 prevents pyroptosis in vascular ECs by improving mitochondrial function.

### Impact of SIRT3 inhibition on NLRP3 inflammasome activation and pyroptosis under simulated microgravity

**Figure 4:**
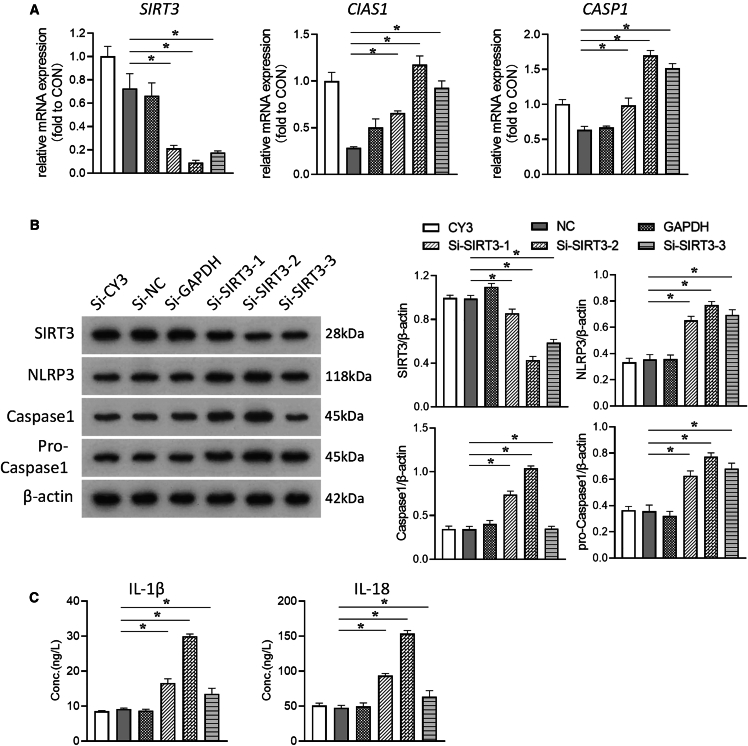
SIRT3 knockdown and NLRP3 inflammasome activation in microgravity-treated HUVECs Three small interfering RNAs targeting SIRT3 (si-SIRT3-1, si-SIRT3-2, and si-SIRT3-3), a non-targeting control (si-NC), a fluorescent control for transfection efficiency (si-CY3), and a positive control for RNAi efficacy (si-GAPDH) were transfected into HUVECs after 48 h. (A) The mRNA expression levels of *SIRT3*, *CIAS1*, an*d CASP1*. (B) The protein expression levels of SIRT3, NLRP3, caspase-1, and pro-caspase-1. (C) Concentration of IL-1β and IL-18 in HUVECs. Mean ± SEM; *n* = 3 biological samples per group in triplicate were measured. Student’s t test: ***∗****p* < 0.05 vs. si-NC.

**Figure 5:**
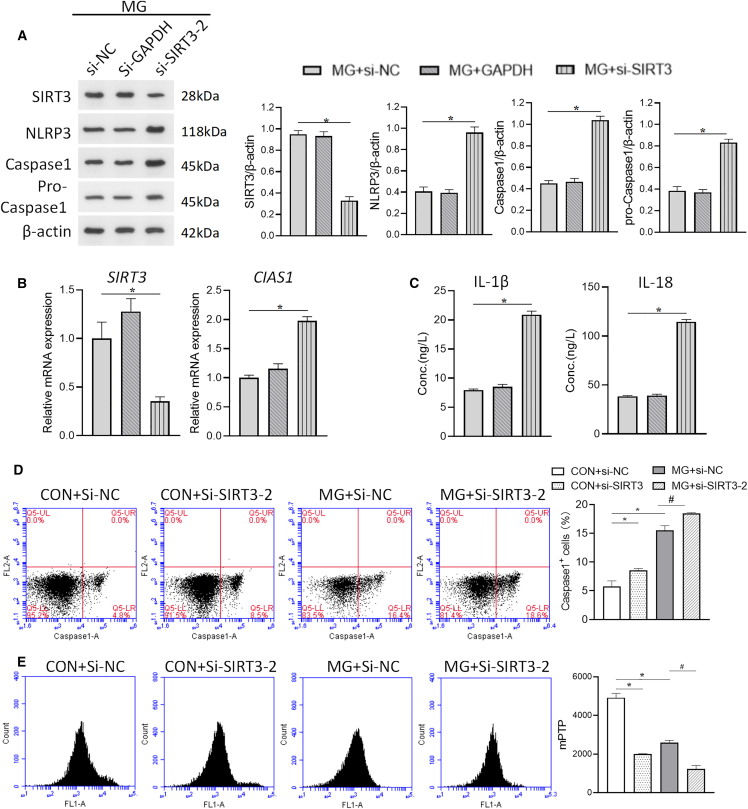
Impact of SIRT3 knockdown on NLRP3 inflammasome activation and pyroptosis in microgravity-treated HUVECs The non-targeting control (si-NC), a positive control for RNAi efficacy (si-GAPDH), and a small interfering RNA targeting SIRT3 (si-SIRT3-2) were transfected into HUVECs 48 h before clinorotation. (A) Representative images displaying the protein expression of SIRT3, NLRP3, caspase-1, and pro-caspase-1, along with semi-quantitative densitometry. (B) The mRNA expression levels of *SIRT3* and *CIAS1*. (C) Concentration of IL-1β and IL-18 in HUVECs. Mean ± SEM; *n* = 3 biological samples per group in triplicate were measured. Student’s t test: ***∗****p* < 0.05 vs. si-NC. (D) The percentage of caspase-1-positive cells. (E) The JC-1 red fluorescence. Mean ± SEM; *n* = 3 biological samples per group in triplicate were measured. Tukey’s HSD test: ***∗****p* < 0.05 vs. CON+si-NC, ^***#***^*p* < 0.05 vs. MG + si-NC.

To further confirm the role of SIRT3 in the regulation of NLRP3 inflammasome and pyroptosis, HUVECs were transfected with siRNA targeting SIRT3 (si-SIRT3). Protein and mRNA levels of SIRT3 were significantly downregulated compared with controls (Figures 4A and 4B), with corresponding upregulation of NLRP3 and caspase-1 protein and mRNA levels (Figures 4A and 4B). The secretion of IL-1β and IL-18 was also significantly increased (Figure 4C). In addition, si-SIRT3-2 had the highest interference efficiency among the three siRNA expression plasmids, and thus si-SIRT3-2 was used to knock down SIRT3. As shown in Figures 5A and 5B, si-SIRT3-2 significantly decreased the protein and mRNA levels of SIRT3 compared with the control, while the protein and mRNA levels of NLRP3 and caspase-1 were significantly increased. The secretion of IL-1β and IL-18 was also further increased (Figure 5C). In addition, cellular ROS was increased and ΔΨm was decreased under simulated microgravity, and these changes were further exacerbated by si-SIRT3-2 (Figures 5D and 5E). The above data suggest that SIRT3 protects HUVECs from pyroptosis under simulated microgravity.

### SIRT3 deficiency and metabolic reprogramming under simulated microgravity

**Figure 6:**
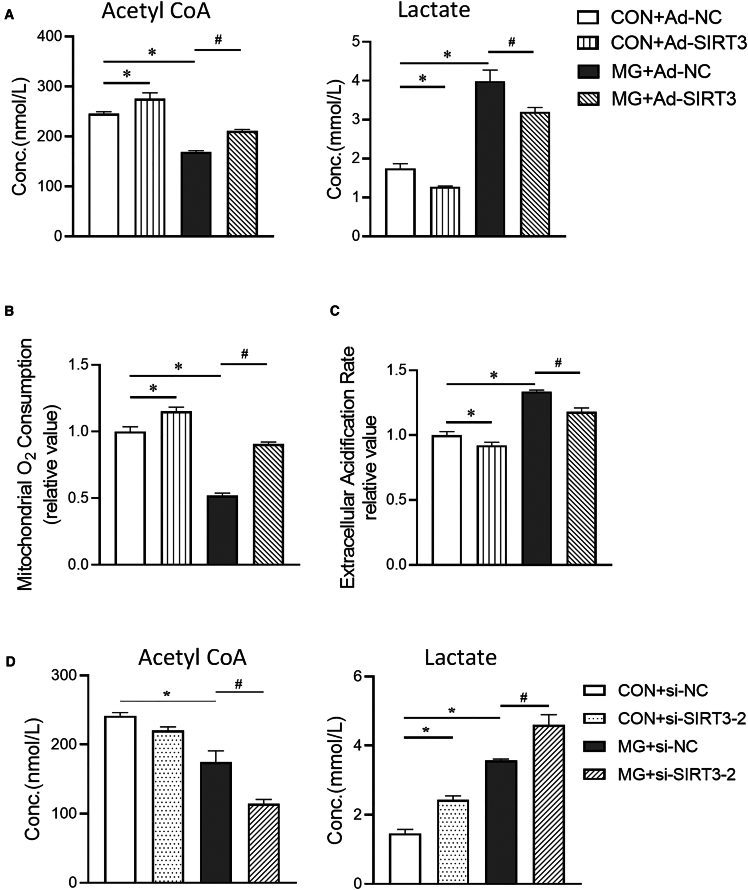
Metabolic phenotype in SIRT3-deficient HUVECs under simulated microgravity (A) The concentration of acetyl-CoA and lactate in HUVECs exposed to microgravity and transfected with Ad-NC or Ad-SIRT3. (B) Oxygen consumption rates in HUVECs exposed to microgravity and transfected with Ad-NC or Ad-SIRT3. (C) Extracellular acidification rates in HUVECs exposed to microgravity and transfected with Ad-NC or Ad-SIRT3. (D) The concentration of acetyl-CoA and lactate in HUVECs exposed to microgravity and transfected with Si-NC or si-SIRT3-2. Mean ± SEM; *n* = 5 biological samples per group in triplicate were measured. Tukey’s HSD test: ***∗****p* < 0.05 vs. CON+Ad-NC or CON+si-NC; ^***#***^*p* < 0.05 vs. MG + Ad-NC or MG+si-NC.

The relationship between the metabolic state and the phenotype of ECs has been well elucidated.^27^ Metabolic switching in ECs drives them into a pro-inflammatory state. To investigate whether SIRT3 regulates metabolic reprogramming, we measured acetyl-CoA and lactate levels and determined oxygen consumption and extracellular acidification rate (ECAR). As shown in Figure 6A, after clinorotation, the concentrations of acetyl-CoA and lactate were decreased and increased, respectively, in HUVECs. Consistent with this, oxygen consumption was decreased and ECAR was increased (Figures 6B and 6C). Overexpression of SIRT3 reversed these phenomena (Figures 6A–6C). When SIRT3 was knocked down, the decrease in acetyl-CoA and the increase in lactate were further exacerbated after clinorotation (Figure 6D), suggesting that SIRT3 deficiency contributes to a shift in metabolism toward a higher level of glycolysis under simulated microgravity.

### Regulation of PDH activity by SIRT3 via PDHA1-K83 acetylation in HUVECs

**Figure 7:**
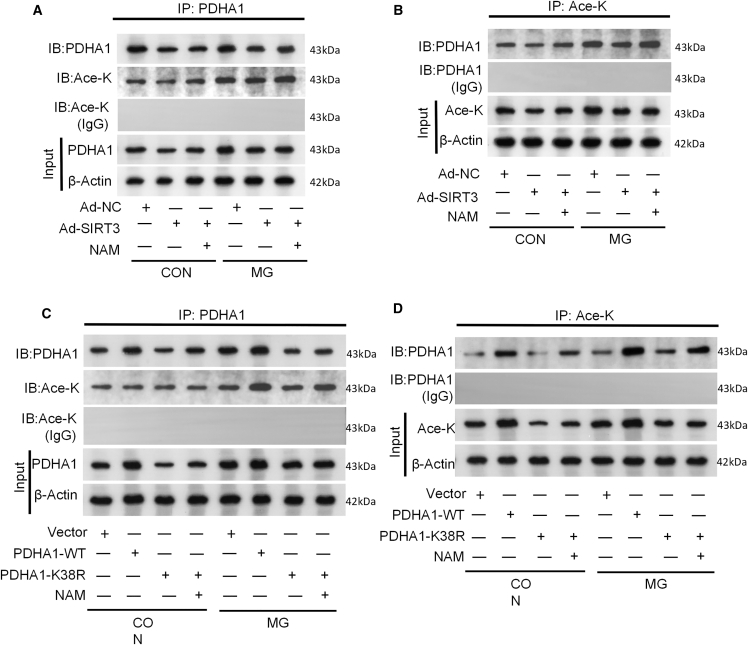
Interaction between SIRT3 and PDHA1 at K83 in microgravity-treated HUVECs (A) Representative western blot images of HUVECs transfected with Ad-NC and Ad-SIRT3 and treated with NAM, immunoprecipitated (IP) with a PDHA1 antibody, and immunoblotted with Ace-K and PDHA1 antibodies. Input PDHA1 and β-actin are also shown. (B) Representative western blot images of cells transfected with Ad-NC and Ad-SIRT3, along with NAM treatment, IP with Ace-K antibody, and immunoblotted with PDHA1 antibody. Inputs for Ace-K and β-actin are also presented. (C) Representative western blot images of HUVECs transfected with vector, PDHA1-WT, and PDHA1-K83R, along with NAM treatment, are shown. The cells were IP with a PDHA1 antibody and subsequently immunoblotted with antibodies against Ace-K and PDHA1. Input PDHA1 and β-actin are also presented. (D) Representative western blot images of HUVECs transfected with vector, PDHA1-WT, and PDHA1-K83R, along with NAM treatment, IP with Ace-K antibody, and subsequently immunoblotted with PDHA1 antibody. Input Ace-K and β-actin are also presented. *n* = 3 biological samples per group.

PDH is a multi-enzyme complex that converts pyruvate to acetyl-CoA, thereby linking glycolysis to the Krebs cycle. SIRT3 regulates PDH activity by deacetylating PDHA1.^28^^,^^29^^,^^30^ To determine whether SIRT3 regulates PDHA1 acetylation, proteins were co-precipitated with PDHA1 antibody (Figure 7A) or acetyl-lysine (Ace-K) antibody (Figure 7B), and samples were then pulled down by immunoblotting with PDHA1 and Ace-K antibodies. Acetylated PDHA1 (Ac-PDHA1) levels were upregulated after clinorotation, SIRT3 overexpression decreased acetylated PDHA1 levels, and the sirtuin inhibitor nicotinamide (NAM) exacerbated acetylated PDHA1 levels (Figures 7A and 7B). A previous study showed that K83 of PDHA1 is associated with the activation of metabolic shifts in the NLRP3 inflammasome in macrophages.^28^ Therefore, a K-to-R mutant at PDHA1 K83 was constructed, and HUVECs were transfected with PDHA1-WT and PDHA1-K83R, treated with NAM, and then immunoprecipitated with antibodies against PDHA1 and Ace-K. As shown in Figures 7C and 7D, the acetylation level of the K83 arginine mutant was lower than that of the wild type (WT) and NAM slightly increased the acetylation level of the K83 arginine mutant under simulated microgravity conditions. The acetylation level of WT was higher than that of vector, which may be attributed to the acetylation of both endogenous and exogenous PDHA1 under simulated microgravity.

### SIRT3-PDH axis in modulating endothelial NLRP3 inflammasome under simulated microgravity

**Figure 8:**
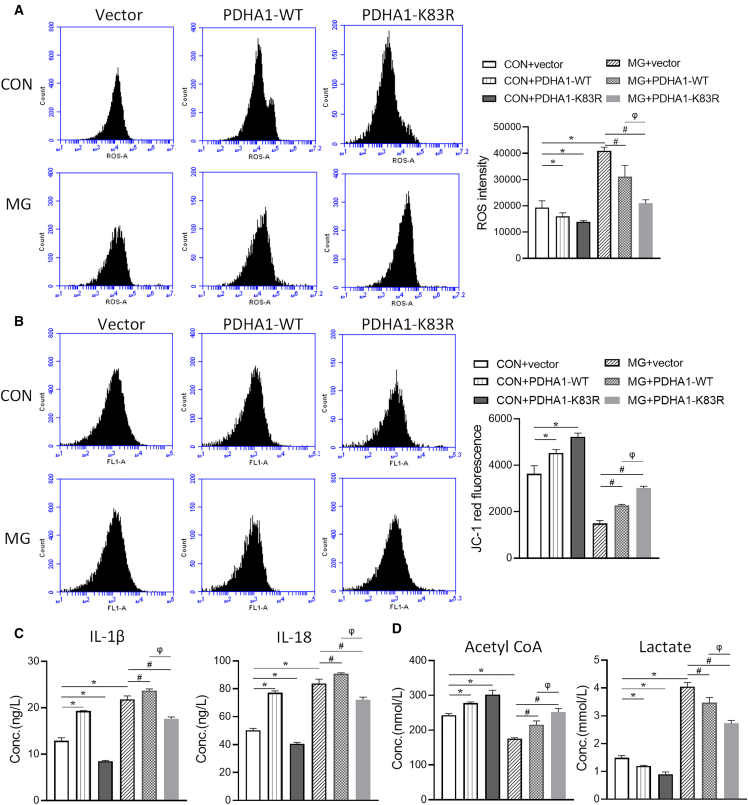
Role of PDHA1 in NLRP3 inflammasome activation and metabolism in microgravity-treated HUVECs HUVECs transfected with vector, wild-type PDHA1 (PDHA1-WT), and lysine 83 K-to-R mutant PDHA1 (PDHA1-K83R), and then exposed to clinorotation 72 h. (A)The cellular ROS level. (B)The JC-1 red fluorescence. (C)The concentrations of IL-1β and IL-18. (D)The concentration of acetyl-CoA and lactate. *n* = 3 biological samples per group in triplicate were measured. ***∗****p* < 0.05 vs. CON + vector, ^***#***^*p* < 0.05 vs. MG + vector, ^*φ*^*p* < 0.05 vs. MG + PDHA1.

**Figure 9:**
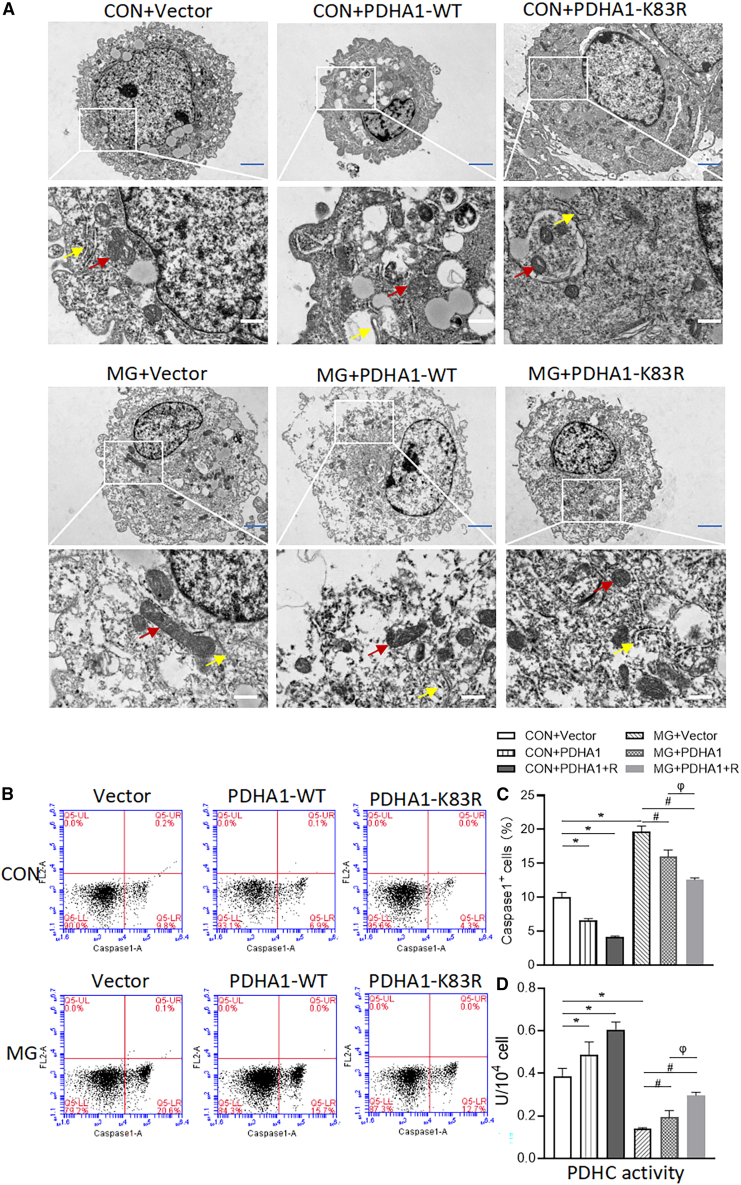
Role of PDHA1 in pyroptosis and PDHC activity in microgravity-treated HUVECs HUVECs transfected with vector, wild-type PDHA1 (PDHA1-WT), and lysine 83 K-to-R mutant PDHA1 (PDHA1-K83R) and then exposed to clinorotation 72 h. (A) Representative TEM images depicting the cellular ultrastructure (red arrows highlighting mitochondria and yellow arrows indicating the endoplasmic reticulum). Blue scale bars, 2 μm; white scale bars, 500 nm. (B and C) The percentage of caspase-1-positive cells. (D) PDHC activity. *n* = 3 biological samples per group in triplicate were measured. ***∗****p* < 0.05 vs. CON + Vector, ^***#***^*p* < 0.05 vs. MG + Vector, ^*φ*^*p* < 0.05 vs. MG + PDHA1.

To further elucidate the role of PDHA1 in NLRP3 inflammasome activation and pyroptosis under simulated microgravity, HUVECs were transfected with PDHA1-WT and PDHA1-K83R. Transfection of PDHA1-WT significantly alleviated oxidative stress (Figure 8A), restored the levels of mitochondrial ΔΨm (Figure 8B), and decreased the levels of IL-1β, IL-18 (Figure 8C), and lactate (Figure 8D). In contrast, transfection of PDHA1-WT significantly restored the level of acetyl-CoA (Figure 8D). Besides, HUVECs in the MG group exhibited morphological features of pyroptosis, such as membrane collapse, cytoplasmic swelling, cytoplasmic vacuolization, and swelling of endoplasmic reticulum and mitochondria, and transfection of PDHA1-WT partially attenuated cellular ultrastructural damage under simulated microgravity (Figure 9A). The percentage of caspase-1-positive cells was significantly decreased after transfection of PDHA1-WT under simulated microgravity (Figures 9B and 9C). Transfection of PDHA1-K83R appeared to have a more potent protective effect, as the mutant form of PDHA1 resisted acetylation under simulated microgravity (Figures 8 and 9). Moreover, PDHC activity correlated with the protective effect after transfection of PDHA1 and PDHA1-K83R (Figure 9D), suggesting that PDHA1 acetylation is involved in the metabolic shift-induced activation of the NLRP3 inflammasome and pyroptosis of ECs under simulated microgravity.

## Discussion

To the best of our knowledge, the results of this study show that under simulated microgravity, reduction of SIRT3 triggers activation of NLRP3 inflammasome by promoting PDHA1 acetylation in ECs. In this process, inhibited PDHA1 activity induces a metabolic shift toward to a higher level of glycolysis, leading to activation of NLRP3 inflammasome. This is the first study to identify the mechanism of metabolic dysfunction in vascular ECs in response to changes in the gravitational unloading.

Microgravity as a special environment leads to a range of health problems, and cardiovascular deconditioning is a key threat to the health of astronauts. Space missions expose astronauts to an increased risk of oxidative stress and inflammatory injury, which can accelerate the progression of cardiovascular diseases.^9^ Our previous studies have shown that phenotypic switching of vascular smooth muscle cells during simulated microgravity exposure plays an important role in vascular remodeling and regulation of vascular homeostasis,^31^^,^^32^ with the key mechanism being mitochondrial dysfunction.^33^^,^^34^^,^^35^ Endothelial dysfunction, an early sign of vascular injury, is characterized by the near-total loss of acetylcholine-induced vasodilation during the Tiangong-2 mission,^36^ and the elevated levels of oxidative stress and inflammation markers in astronauts on the International Space Station, despite no change in vasodilation function.^9^ The NASA twin study further confirmed this phenomenon, showing that the serum levels of pro-inflammatory cytokines (IL-1α, IL-1β, etc.) in the flight group remained consistently higher than those in the ground control group.^37^ In terms of mechanism, Versari et al. studied HUVECs after 10 days of microgravity exposure aboard the International Space Station and found varying degrees of expression of genes involved in cell adhesion, OXPHOS, stress response, cell cycle, and apoptosis and upregulated thioredoxin-interacting protein (TXNIP).^11^ TXNIP increases ROS production and activates NLRP3 inflammasome to promote the maturation and release of IL-1β and IL-18.^38^ Our *in vivo* experiments confirm that NLRP3 inflammasomes exacerbate endothelial inflammation and apoptosis through the caspase-1 pathway.^16^ Previous studies have indicated that microgravity enhances the fluidity of cellular membranes.^39^ The increased membrane fluidity and altered membrane lipid composition may facilitate the abnormal aggregation of NOX subunits, resulting in heightened ROS production and activation of the NLRP3 inflammasome.^40^^,^^41^ Thus, inhibition of the NLRP3 inflammasome is a potential approach to prevent vascular dysfunction.^42^ However, ROS dynamics under microgravity remains controversial. Cazzaniga and Locatelli et al. observed a modest but statistically significant increase in ROS after 2 h of simulated microgravity using the rotating wall vessel, although no further changes were noted afterward.^12^^,^^24^ In the present study, we observed a 2-fold increase in ROS at 72 h of clinorotation in the microgravity group compared to controls. In contrast, clinorotation in suspension cells reduced phagocytosis-mediated ROS production in N8383 cells (rat alveolar macrophages), with a similar reduction seen in parabolic flights under real microgravity conditions.^43^ These discrepancies may stem from variations in clinostat parameters or cell-type specificity. Thus, future studies should systematically investigate clinorotation conditions (e.g., speed, duration) and cell-specific oxidative stress pathways to clarify NLRP3 activation mechanisms, ultimately advancing strategies against microgravity-associated vascular dysfunction.

In diet-induced obese mice, SIRT3 deficiency exacerbates endothelial function as evidenced by impaired insulin- and acetylcholine-dependent endothelial vasodilator function.^44^ Studies have demonstrated that mitochondrial ROS (mtROS), Ca^2+^ overload, mitochondrial DNA release after mitochondrial injury, increased lipid metabolites, and mitochondrial fusion proteins can activate the NLRP3 inflammasome.^26^ Mechanistically, excessive ROS production can induce post-translational modifications of NLRP3, promoting its oligomerization and subsequent assembly with ASC and caspase-1, which ultimately leads to the secretion of pro-inflammatory cytokines such as IL-1β and IL-18.^45^ SIRT3 enhances ROS detoxification through activation of MnSOD and catalase.^46^ Downregulation of SIRT3 increases mtROS generation, accompanied by reduction of NO release and activation of NLRP3 inflammasome, whereas activation of SIRT3 by honokiol inactivates the NLRP3 pathway, thereby preventing endothelial dysfunction.^46^^,^^47^ We reported that SIRT3 mRNA and protein expression in HUVECs were inhibited under simulated microgravity. However, the inhibition level of SIRT3 mRNA is more significant than that of its protein, which may be influenced by post-transcriptional regulatory factors such as translation efficiency, protein degradation, or modification. After exposure to clinorotation, HUVECs showed mitochondrial dysfunction, activation of the NLRP3 inflammasome pathway, and increased pyroptosis, which were partially reversed by SIRT3 overexpression. Therefore, we conclude that under simulated microgravity, SIRT3 inhibition loses its protective effect against endothelial inflammation and mitochondrial dysfunction, leading to a persistent pro-inflammatory and pro-oxidative state.

Despite immediate access to blood oxygen, healthy ECs rely on glycolysis rather than OXPHOS to maintain vascular barrier function, tissue homeostasis, and the ability to respond rapidly to stresses such as hypoxia, nutrient deprivation, or tissue damage.^48^^,^^49^ ECs produce up to 85% of their ATP from glycolysis, comparable to or higher than cancer cells. ECs have the ability to switch from OXPHOS to glycolysis as the primary energy source, known as metabolic flexibility, to protect cells with increased energy demands from oxidative stress. Under pathological conditions, ECs increase glycolytic flux by upregulating PFKFB3.^48^^,^^50^ Glycolysis promotes nuclear factor-κB-driven vascular inflammation, and knockdown of PFKFB3 almost completely blocked all TNF-α-induced release of the proinflammatory cytokines/chemokines.^51^ As ECs are prone to mechanical stress, studies have demonstrated that hemodynamics can influence metabolism of ECs.^52^ Previously, Wu et al. showed that disturbed flow, which occurs in arterial bifurcations and curvatures, induces glycolysis and reduces mitochondrial respiratory capacity in human aortic ECs by activating HIF-1a, which increases glycolytic enzymes and pyruvate dehydrogenase kinase-1 (PDK-1).^53^ And this metabolic switching leads to vascular inflammation. Recently, studies have shown that mitochondrial function is also important for the homeostatic regulation and angiogenic capacity of endothelium.^54^^,^^55^ Previously, the multi-omics data from NASA’s GeneLab suggested that gene expression related to energy generation was changed in HUVECs.^56^ Locatelli et al. showed that after 4 and 10 days of exposure to simulated microgravity, mitochondrial content, oxygen consumption, and maximal respiratory capacity were all simultaneously reduced, suggesting the acquisition of an efficient phenotype to meet the metabolic challenges created by gravitational unloading.^24^ In the present study, we also found a similar alteration in ECs, manifested as reduced oxygen consumption and acetyl-CoA and increased ECAR and lactate. Moreover, evidence of mitochondrial dysfunction was also observed in the NASA Twin Study, as increased levels of lactate were detected in astronaut’s urine and plasma during spaceflight, suggesting a shift from aerobic to anaerobic metabolism.^37^ The lactate/pyruvate ratio, as determined by gas chromatography-tandem mass spectrometry urine metabolomics data, also indicated this metabolic shift.^37^ Additionally, these metabolic changes were associated with increased signs of inflammation in astronaut during spaceflight.^37^

SIRT3 is a well-known mitochondrial protein whose deacetylase activity regulates a variety of metabolic processes. However, the role of SIRT3 in endothelial metabolism remains inadequately explored. In ECs, pyruvate—a glycolysis metabolite—is either converted to lactate in the cytoplasm or transported to the mitochondria by the mitochondrial pyruvate carrier, where it is then oxidatively converted to acetyl-CoA by PDHC. Previously, He et al. found that SIRT3 knockout cells displayed reduced expression and increased acetylation of the glycolytic enzyme PFKFB3, resulting in decreased glycolysis and angiogenesis, along with an elevation of pro-inflammatory factors.^57^ By relying on glycolysis to produce ATP, ECs conserve oxygen, thereby protecting themselves from mitochondrial electron leakage and the generation of ROS.^58^ Furthermore, the absence of SIRT3 leads to an increased rate of oxygen consumption and maximal respiratory capacity in ECs, along with elevated levels of ROS, suggesting a shift from glycolysis to OXPHOS.^57^ However, in pulmonary hypertension, the inflammatory and hyperproliferative endothelial phenotype is linked to metabolic reprogramming that shifts from mitochondrial respiration to glycolysis at even higher levels than those observed in healthy ECs.^59^ This shift is attributed to the upregulation of PDK, which inhibits the activity of PDHC. In the present study, we demonstrate that the downregulation of SIRT3 is involved in the metabolic switch to elevated glycolysis levels and the inhibition of OXPHOS in HUVECs under simulated microgravity. The variations observed between these studies may be attributed to differing pathophysiological conditions and levels of SIRT3 expression. A previous study reported that SIRT3 regulates metabolic pathways in energy-producing and energy-utilizing tissues in distinct ways, suggesting that SIRT3 modulates mitochondrial acetylation in a tissue-specific manner.^60^ The mitochondrial acetylation of primary microvascular ECs in the hearts and lungs of SIRT3 knockout mice may differ from that observed in HUVECs. Moreover, aside from acetylation, the deletion or downregulation of SIRT3 may exert varying effects on the expression of metabolic enzymes. Nonetheless, the present study is the first to identify a functional role for SIRT3 in endothelial metabolism under simulated microgravity.

In vascular ECs, metabolism is closely linked to inflammatory pathways. Recent studies have shown that treatment of ECs with lactate disrupts EC function and induces mesenchymal-like function through activation of the TGF-β/Smad2 pathway following hypoxia.^61^ In lipopolysaccharide-exposed HUVECs and C57BL/6 mice, glycolysis and lactate production are increased in ECs, whereas dichloroacetic acid-mediated activation of PDHC prevents lactate production and protects against barrier dysfunction in HUVECs.^62^ In the present study, we demonstrate that simulated microgravity activates NLRP3 inflammasome and pyroptosis by promoting glycolysis, while impairing OXHPOS in ECs by inhibiting PDHC activity. PDHA1 is a constitutive enzyme of PDHC that catalyzes the conversion of pyruvate to acetyl-CoA, which is subsequently utilized by the citric acid cycle and the OXPHOS to produce ATP. Lactate accumulation resulting from aerobic to anaerobic oxidation suggests that PDHA1 acetylation plays a crucial role in regulating PDHC activity and energy metabolism.^63^ Accumulated lactate has been reported to activate NLRP3 inflammasome in macrophages, as blocking the conversion of pyruvate to lactate reduces NLRP3 and caspase-1 expression.^28^^,^^64^ Previous studies have shown that activation of NLRP3 inflammasomes requires PKR phosphorylation^65^ and that lactate-dependent PKR phosphorylation is responsible for glycolysis-induced activation of NLRP3 inflammasomes.^64^ Consistent with this, our study also showed that increased NLRP3 inflammasome activation was accompanied by an increase in cellular lactate and overexpression of PDHA1 significantly inhibited lactate production and NLRP3 inflammasome activation. Previously, SIRT3 was found to deacetylate lysine 321 (K321) of PDHA1 in cancer cells,^27^ K336 in skeletal muscle,^63^ and K83 in renal tubular epithelial cells and adipocytes.^29^^,^^30^ SIRT3-mediated deacetylation of the above lysine residues increased PDH activity and promoted mitochondrial oxidation. Tong et al. found that SIRT3 deacetylation of K83 of PDHA1 inhibited NLRP3 inflammasome activation by regulating metabolic shifts.^64^ In the present study, we confirmed that SIRT3 deacetylated the K83 site of PDHA1 in HUVECs exposed to simulated microgravity. In addition, overexpression of SIRT3 reversed the anaerobic metabolism of ECs, which was associated with a reduction in lactate content and inhibition of pyroptosis, suggesting that SIRT3 protects the mitochondrial function of ECs under simulated microgravity conditions and prevents pyroptosis by deacetylating PDHA1.

This study reports the mechanism by which simulated microgravity induces endothelial inflammation and pyroptosis in HUVECs. Under simulated microgravity, SIRT3 downregulation leads to mitochondrial dysfunction, NLRP3 inflammasome activation, and caspase-1 cleavage in HUVECs. Decrease in SIRT3 increased the acetylation level of PDHA1 lysine 83 and shifted the endothelial metabolic phenotype to a higher level of glycolysis. Overexpression of SIRT3 and PDHA1 reversed the metabolic phenotypic shift and suppressed the inflammatory phenotype. Therefore, we propose that SIRT3 is a potential therapeutic target for attenuating NLRP3 inflammation-associated inflammation and restoring normal cellular metabolism in ECs under simulated microgravity.

### Limitations of the study

This study has several limitations. First, while we measured acetyl-CoA and lactate levels to assess glycolysis, we acknowledge that acetyl-CoA is a central metabolite involved in various pathways, including the citric acid cycle, fatty acid synthesis, and ketogenesis. Its levels are influenced by several factors, including mitochondrial function, AMP-activated protein kinase (AMPK), fatty acid metabolism, as well as other sirtuins such as SIRT1 and SIRT2.^66^ This complexity means we cannot fully exclude the influence of other metabolic pathways on our findings. Additionally, our investigation focused on the expression and regulatory mechanisms of the SIRT3/PDH axis after 72 h of clinorotation. Further studies are necessary to incorporate multiple time points to provide a more comprehensive understanding of cellular dynamics over time. Furthermore, future studies in animals or humans are needed to gain a deeper understanding of the *in vivo* implications, and more reference genes or proteins are needed.

## Resource availability

### Lead contact

Further information and requests for resources and reagents should be directed to and will be fulfilled by the lead contact, Ran Zhang (zhangran@plagh.org).

### Materials availability

The virus strains and cell line used in this study will be available upon request; however, we may require payment and/or a completed materials transfer agreement if there is potential for commercial application.

### Data and code availability

• All data associated with this study are present in the paper or the supplemental information.
• This paper does not report any original code.
• Any additional information required to reanalyze the data reported in this paper is available upon request from the lead contact.

## Acknowledgments

This work was supported by the Medical Science and Technology Youth Cultivation Program (grant no. 21QNPY133) and the 10.13039/501100001809National Natural Science Foundation of China (grant nos. 82171857 and 82302114).

## Author contributions

R.Z., X.L., and M.J. conceived and designed the experiments. M.J., J.S., K.L., L.L., and S.Y. performed the experiments. J.Z. and S.S. performed data analysis. R.Z., M.J., and J.S. wrote the manuscript. All the authors have full access to all the data in current study and final responsibility for the decision to submit for publication.

## Declaration of interests

The authors declare that they have no conflict of interest.

## STAR★Methods

### Key resources table

**Table:** 

REAGENT or RESOURCE	SOURCE	IDENTIFIER
**Antibodies**		

Anti-SIRT3 Rabbit antibody	Abcam	Cat. # ab189860
Anti-NLRP3 Rabbit antibody	Abcam	Cat. # ab263899
Anti-Caspase-1 Rabbit antibody	Abcam	Cat. # ab238979
Anti-pro-Caspase-1 Rabbit antibody	Abcam	Cat. # ab238972
Anti-β-actin Mouse antibody	Abcam	Cat. # ab8226
Normal Mouse IgG Polyclonal Antibody	Millipore	Cat. # 12-371
Anti-PDHA1 Rabbit antibody	Abcam	Cat. # ab168379
Anti-acetyl Lysine Rabbit antibody	Abcam	Cat. # ab190479
Anti-Caspase-1 antibody	Proteintech	Cat. # 81482-1-RR
HRP-labeled Goat Anti-Rabbit IgG(H+L)	Beyotime	Cat. # A0208
HRP-labeled Goat Anti-Mouse IgG(H+L)	Beyotime	Cat. # A0216
Goat anti-Rabbit IgG (H+L) Cross-Adsorbed Secondary Antibody, Alexa Fluor 594	Thermo Scientific	Cat. # A-11012

**Bacterial and virus strains**		

Ad-SIRT3	Regete Biological Technology	N/A
Ad-NC	Regete Biological Technology	N/A
si-SIRT3-1	Regete Biological Technology	N/A
si-SIRT3-2	Regete Biological Technology	N/A
si-SIRT3-3	Regete Biological Technology	N/A
si-NC	Regete Biological Technology	N/A
si-CY3	Regete Biological Technology	N/A
si-GAPDH	Regete Biological Technology	N/A
PDHA1-WT	Regete Biological Technology	N/A
PDHA1-K83R	Regete Biological Technology	N/A

**Chemicals, peptides, and recombinant proteins**		

Protein G-Agarose	Roche	Cat. #11719416001
Epoxy Embedding Medium Kit	Sigma-Aldrich	Cat. # 45359
Mitochondrial membrane potential assay kit with JC-1	Beyotime	Cat. # C2006
Extracellular Oxygen Depletion Assay Kit	Abcam	Cat. # ab197242
Extracellular Acidification Rate Assay Kit	BestBio	Cat. # BB-48311
Lactic Acid assay kit	Jiancheng Bioengineering Institute	Cat. # A019-2-1
Pyruvate Dehydrogenase Assay Kit	Solarbio	Cat. # BC0385

**Critical commercial assays**		

RevertAid™ First Strand cDNA Synthesis Kit	Thermo Scientific	Cat. # K1622
Mycoplasma PCR Detection Kit	Beyotime	Cat. # C0301S
SYBR® Premix Ex Taq TM	Takara	Cat. # RR420A
Human IL-1β ELISA Kit	Jingmei Biotechnology	Cat. #JM-03336H1
Human IL-18 ELISA Kit	Jingmei Biotechnology	Cat. #JM-03294H1
Human acetyl coenzyme A ELISA kit	Enzyme-linked Biotechnology	Cat. # ml057617
ROS Fluorescent Probe-DHE	Vigorous Biotechnology	Cat. # R001

**Experimental models: Cell lines**		

HUVEC	BLUEFBIO™	Cat. # FN607200285

**Oligonucleotides**		

*SIRT3* Forward:TGGCATTCCAGACTTCAG Reverse:CGTTGGGCTTGTAGTTTC	This paper	N/A
*NLRP3* Forward:CTGGAGGATGTGGACTTG Reverse:GTCTGCCTTCTCTGTCTG	This paper	N/A
*CASP1* Forward:TGACATCACAGGCATGAC Reverse:CTTCCCGAATACCATGAGAC	This paper	N/A
*ACTB* Forward:CATCGTCCACCGCAAATGCTTC Reverse:AACCGACTGCTGTCACCTTCAC	This paper	N/A

**Software and algorithms**		

Image J	National Institutes of Health	https://imagej.net/ij/
ABI 7300 Real-Time PCR System	Thermo Scientific	https://www.thermofisher.cn/cn/zh/home/technical-resources/software-downloads/applied-biosystems-7300-real-time-pcr-system.html
FlowJo 7.6	BD Biosciences	https://flowjo.com/
GraphPad Prism 9.0	GraphPad	https://www.graphpad.com/

### Experimental model and study participant details

#### Cell culture and drug treatments

HUVECs were purchased from BLUEFBIO (Shanghai, China) and cultured in Dulbecco's Modified Eagle Medium (Hyclone, Logan, UT, USA) supplemented with 10% fetal bovine serum (Gibco, Grand Island, NY, USA), 0.05 mg/ml endothelial cell growth supplement, 0.1 mg/ml heparin, 100 units/ml penicillin and 100 mg/ml streptomycin (Sigma-Aldrich, St. Louis, MO, USA) at 37°C within 95% humidified air and 5% CO_2_. The cells were routinely assessed for the expression of endothelial markers and were used for 6 to 8 passages. The SIRT3 inhibitor, nicotinamide, was obtained from Sigma-Aldrich (St. Louis, MO, USA) and were used at a concentration of 10 mmol/L. These cell lines have been authenticated with the STR genotype testing by the supplier. All the cell lines were routinely tested for mycoplasma contamination using the Mycoplasma PCR Detection Kit (Beyotime, Shanghai, China) and consistently found to be negative.

### Method details

#### Ground-based simulation of microgravity

Two-dimensional clinorotation is an effective way to simulate microgravity in cells.^67^^,^^68^ HUVECs were seeded onto a 2.55×2.15 cm (5.48 cm^2^) coverslip at a density of 1×10^5^ and cultured in a 6-well plate for 24 hours to ensure proper cell adhesion. Subsequently, the coverslip was transferred to a custom stainless-steel holder and positioned inside a polycarbonate culture vessel, 12.5 mm from the rotating axis. The experimental group was subjected to rotation horizontally at 30 rpm in a constant temperature of 37°C. Under these conditions, HUVECs no longer cannot perceive gravity and, thus, experience simulated microgravity. The control group was kept in static culture within the same incubator, utilizing the same medium as mentioned previously, and ensuring that all culture vessels were meticulously filled and degassed. Temperature variations (±0.5°C) and fluid coupling status were monitored throughout the experiment to maintain the stability and reproducibility of the experimental conditions. After clinorotation, the HUVECs were harvested immediately for analysis.

#### Western blot analysis

HUVECs were rinsed with 4°C PBS and scraped in RIPA lysis buffer (Beyotime, Shanghai, China) containing 1 mM phenylmethylsulfonyl fluoride (PMSF). Protein content was determined using a BCA protein assay kit (Thermo Scientific, Rockford, IL, USA). Extracts were fractionated by SDS-PAGE and transferred to polyvinylidene difluoride (PVDF) membranes (Thermo Fisher). The membranes were incubated with antibodies against SIRT3 (ab189860, 1:500), NLRP3 (ab263899, 1:500), Caspase-1 (ab238979, 1:1000), pro-Caspase-1 (ab238972, 1:1000) and β-actin (ab8226, 1:1500). These antibodies were obtained from Abcam (Cambridge, MA, USA). Then the membranes were incubated with HRP-conjugated secondary antibody (A0208, Beyotime, Shanghai, China) for 2 h. Blots were washed and developed with the ECL system (Millipore). The densities of the bands were analyzed with Image J software (NIH, USA).

#### Immunoprecipitation and immunoblotting

HUVECs were rinsed with 4°C PBS and scraped in RIPA lysis buffer (Beyotime, Shanghai, China) containing protease inhibitor. For immunoprecipitation, appropriate control IgG (12-371, Millipore, MA, USA) was added, and the lysate was precleared with protein G-Agarose beads (11719416001, Roche, Basel, Switzerland) and incubated for 1 hour at 4°C. PDHA1 antibody (ab168379, 1:1000, Abcam) or acetyl lysine antibody (ab190479, 1:500, Abcam) was added and incubated overnight at 4°C with shaking. Protein G-agarose beads were added and incubated at 4°C for 4 hours. The immunoprecipitant was collected by centrifugation and the supernatant was discarded. The pellet was washed four times with PBS buffer, and the bound proteins were eluted by boiling with SDS sample buffer, resolved by SDS-PAGE, and then analyzed by western blot analysis.

#### Quantitative polymerase chain reaction (qPCR)

HUVECs were scraped and homogenized with Trizol (Thermo Scientific, Waltham, MA, USA) and reverse transcribed into cDNA with RevertAid™ First Strand cDNA Synthesis Kit (K1622, Thermo Scientific). Quantitative real-time polymerase chain reaction (qPCR) was performed with SYBR® Premix Ex TaqTM (RR420A, Takara, Shiga, Japan) on an ABI 7300 Real-Time PCR System (Thermo Scientific). The PCR reaction mixture consisted of 10 μL SYBR Green Mix, 1 μL forward primer (10 μM), 1μL reverse primer (10 μM), 11 μL ddH_2_O and 2 μL cDNA. The amplification protocol consisted of an initial denaturation at 95°C for 10 min, followed by 40 cycles of denaturation at 95°C for 15 seconds and annealing at 55°C for 45 seconds. A melting curve analysis was subsequently performed with 95°C for 15 seconds, 60°C for 1 min, then gradual heating from 60°C to 95°C to assess reaction specificity. Gene expression was analyzed using the 2^−ΔΔCt^ method, normalizing target gene Ct values to the β-actin (ΔCt= Ct_target gene_ − Ct_β-actin_). Relative expression (fold change) was calculated as 2^−ΔΔCt^, where ΔΔCt = ΔCt(sample) − ΔCt(control).

**Table:** Primers used for PCR

	Forward primer	Reverse primer
*SIRT3*	TGGCATTCCAGACTTCAG	CGTTGGGCTTGTAGTTTC
*NLRP3*	CTGGAGGATGTGGACTTG	GTCTGCCTTCTCTGTCTG
*CASP1*	TGACATCACAGGCATGAC	CTTCCCGAATACCATGAGAC
*ACTB*	CATCGTCCACCGCAAATGCTTC	AACCGACTGCTGTCACCTTCAC

#### Enzyme-linked immunosorbent assay (ELISA)

After clinorotation, the medium was transferred to a centrifuge tube and centrifuged at 1500 rpm for 10 min at 4°C and the supernatant was collected. The concentrations of IL-1β, IL-18 and acetyl-CoA in the culture medium were determined by commercially available ELISA Kits for IL-1β, IL-18 (Jingmei Biotechnology, Jiangsu, China) and acetyl-CoA (Enzyme-linked Biotechnology, Shanghai, China). The protocols for all of these assays were followed according to the manufacturer instructions. All assays were performed in triplicate at room temperature.

#### Flow cytometry analysis of pyroptosis

HUVECs were harvested using 0.25% trypsin-EDTA for 5 min at 37°C and neutralized with serum-containing medium, followed by centrifugation at 300×g for 5 min and two washes with ice-cold PBS. For intracellular staining, cells were fixed with 4% PFA for 15 min and permeabilized with 0.1% Triton X-100 for 10 min at room temperature, with PBS washes between steps. After blocking with 5% BSA/PBS for 30 min at 4°C, cells were incubated with primary anti-Caspase-1 antibody (81482-1-RR, 1:100 dilution, 5 μg/mL) for 2 h at 4°C, washed, and then stained with Alexa Fluor 594-conjugated secondary antibody (1:500 dilution) for 1 h at 4°C. Samples were analyzed flow cytometry within 1 h using a FACScan instrument (BD Biosciences, Mountain View, CA). Data were analyzed with FlowJo 7.6 software (BD Biosciences, Franklin Lakes, NJ, USA).

#### Assay of cellular oxidative stress

HUVECs were harvested using 0.25% Trypsin (EDTA free) and resuspended in 4°C PBS after 72 h of clinorotation. Cells were incubated with 50μM dihydroethidium (DHE, Vigorous Biotechnology, Beijing, China) for 30 min in the dark at 37°C. After incubation, cells were washed with pre-cooled PBS and resuspended in 0.5 ml of cold PBS. The red fluorescence of oxidative stress was detected using the FL2 channel of a BD flow cytometer. The excitation and emission wavelengths are 480 nm and 590 nm, respectively. Data were analyzed with FlowJo 7.6 software (BD Biosciences, Franklin Lakes, NJ, USA).

#### Transmission electron microscopy

HUVECs from both the control and clinorotation groups were scraped, harvested, and centrifuged at 1500 rpm for 10 min. The cell pellets were fixed in 2.5% glutaraldehyde at 4°C for 2 hours. After glutaraldehyde, cells were rinsed in buffer and incubated in 1% OsO_4_ for 2 hours at 4°C. The samples were then dehydrated with graded alcohol and acetone at room temperature, embedded in epoxy resin (Epon812, HEAD , Warrington, PA, USA), and sectioned as slices with a thickness of 60 nm by the ultramicrotome (EM UC7, Leica, Germany). After staining with 3% uranyl acetate and lead citrate, the ultrastructure of the HUVECs was examined using a transmission electron microscope (HT7700, Hitachi, Japan).

#### SIRT3 overexpression and knockdown

SIRT3 was overexpressed by transient transfection of SIRT3-expressing adenovirus (Ad-SIRT3). The Ad-SIRT3 and its non-targeting sequences (negative controls, Ad-NC) were purchased from Regete Biological Technology Co., Ltd (Beijing, China). HUVECs at 75% confluence were transfected with Ad-SIRT3 and the transfection efficiency was determined by western blot analysis and qPCR.

SIRT3 was knocked down by RNA interfering technology. Three small interfering RNA targeting SIRT3 (si-SIRT3-1, si-SIRT3-2 and si-SIRT3-3) were constructed (Regete Biological Technology Co., Ltd, Beijing, China). Si-NC was served as negative control with no homology to the target gene sequence. The fluorescence-labeled negative control (si-CY3) with no homology to the target gene sequence was used to test transfection efficiency. And si-GAPDH was served as a positive control. HUVECs at 75% confluence were transfected with siRNAs and the protein expression was determined by Western blot analysis.

#### JC-1 staining

JC-1 is a cationic dye for studying the membrane potential of mitochondria. JC-1 (Beyotime, Shanghai, China) was diluted to prepare a JC-1 working solution according to the manufactures’ instructions. After clinorotation, HUVECs were harvested using 0.25% Trypsin (EDTA free) and washed with PBS, and the concentration was adjusted to 1x10^6^/mL. Then, 0.5 mL of JC-1 working solution were added into 0.5 mL of cell suspension. Cells were incubated at 37°C for 20 min. The positive control group was treated with 10μM CCCP (Carbonyl cyanide 3-chlorophenylhydrazone) simultaneously. After incubation, the cell suspension was centrifuged and the supernatant was discarded. After washing the cells twice with pre-cooled JC-1 staining buffer, they were suspended in 0.5 ml of buffer. When the mitochondrial membrane potential is high, JC-1 aggregates in the mitochondrial matrix, forming J-aggregates that emit red fluorescence. The red fluorescence of JC-1 polymers was detected using the FL2 channel of a BD flow cytometer. The maximum excitation and emission wavelengths of these J-aggregates are 525 nm and 590 nm, respectively. When cells undergo apoptosis, the mitochondrial membrane potential decreases, resulting in a reduction in red fluorescence intensity.

#### Extracellular oxygen consumption assay

After 72 h of clinorotation, HUVECs were harvested using 0.25% Trypsin (EDTA-free) and seeded in a 96-well plate at a density of 5×10^5^ cells/well. The rate of extracellular oxygen consumption of HUVECs was measured using the Extracellular Oxygen Depletion Assay Kit (ab197242, Abcam, Cambridge, UK) according to the manufacturer's instructions. Fluorescence intensity was analyzed using a multifunctional microplate reader (Tecan, Männedorf, Switzerland) preheated at 37°C. The extracellular oxygen consumption signal was measured at 1.5-minute intervals for 120 minutes at Ex/Em = 380/650 nm.

#### Extracellular acidification rate assay

After 72 h of clinorotation, HUVECs were harvested using 0.25% Trypsin (EDTA-free) and inoculated into a 96-well plate at a density of 8×10^4^ cells per well. The rate of extracellular acidification rate of HUVECs was measured using the Extracellular Acidification Rate Assay Kit (BB-48311, BestBio, Nanjing, China) according to the manufacturer's instructions. Fluorescence intensity was analyzed using a multifunctional microplate reader (Tecan, Männedorf, Switzerland) preheated at 37°C. The extracellular acidification signal was measured at 3-minute intervals for 120 minutes at Ex/Em = 488/580 nm.

#### Lactate assay

The culture medium was collected and mixed with enzyme and color developing agent according to the manufacturer's instructions (A019-2-1, Jiancheng Bioengineering Institute, Nanjing, China). Then, the mixture was incubated at 37°C for 0.5h. The absorbance was determined by ultraviolet and visible spectrophotometer (Lab-Spectrum, Shanghai, China). The lactate concentrations were given by $\frac{A_{sample}-A_{blank}}{A_{standard}-A_{blank}}\times C_{standard}÷Cpr$, where *C*_*standard*_ = 3*mmol*/L is the concentration of the standard, *Cpr* is the protein concentration, and *A* is absorbance.

#### PDHC activity assay

The activity of the PDH complex was measured using a Pyruvate Dehydrogenase Assay Kit (BC0385, Solarbio, Beijing, China). The cell lysis was mixed with the assay agent, and the mixture was incubated at 37°C for 10min. The absorbance was measured at 605 nm, and PDHC activity was calculated according to the manufacturer's instructions.

### Quantification and statistical analysis

The data are expressed as the mean ± SEM. GraphPad Prism 9.0 (GraphPad Software Inc., San Diego, CA, USA) was used for all statistical analyses and figure presentation. Statistical evaluation was performed using 2-tailed paired Student’s t test or a one-way ANOVA followed by the Turkey’s post hoc test. Error bars indicate the SEM throughout.A *P* value < 0.05 was considered statistically significant. All the statistical details can be found in the figure legends.
