# Supplementary material for: SIRT3 regulates PDHA1 acetylation in HUVECs to modulate inflammation and pyroptosis under clinorotation
Source: iScience. 2025 Oct 16;28(11):113790. doi: 10.1016/j.isci.2025.113790 (PMC12663655; doi:10.1016/j.isci.2025.113790)

iScience, Volume *28*

Supplemental information

SIRT3 regulates PDHA1 acetylation in HUVECs to modulate inflammation and pyroptosis under clinorotation

Min Jiang, Junjie Shao, Kun Lin, Lejian Lin, Shuai Yue, Haojie Yan, Jingjing Zhou, Shujin Shi, Xin Li, and Ran Zhang

Data S1: Original images of western blots.

Fig 1A

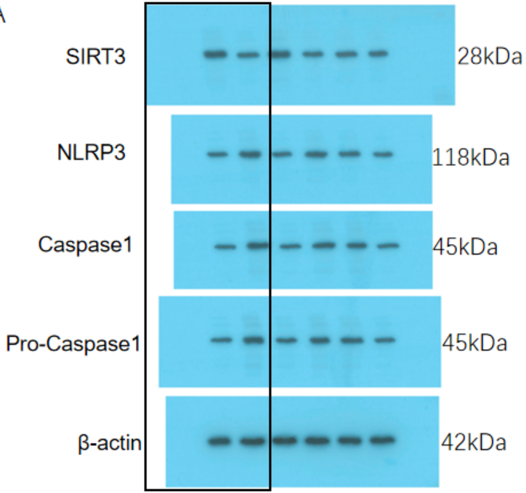

Fig 2C

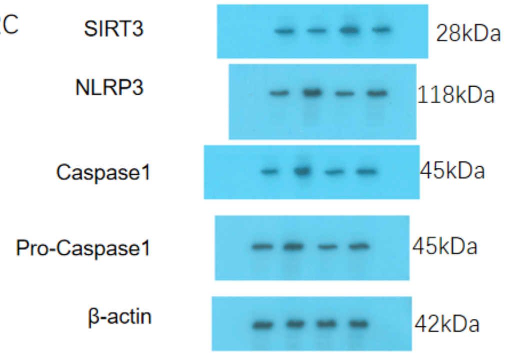
SIRT3
28kDa
NLRP3
118kDa
Caspase1
45kDa
Pro-Caspase1
45kDa
β-actin
42kDa

Fig 4B

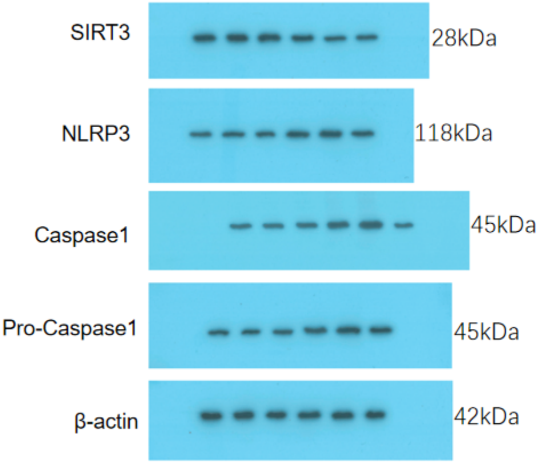
SIRT3
28kDa
NLRP3
118kDa
Caspase1
45kDa
Pro-Caspase1
45kDa
β-actin
42kDa

Fig 5A

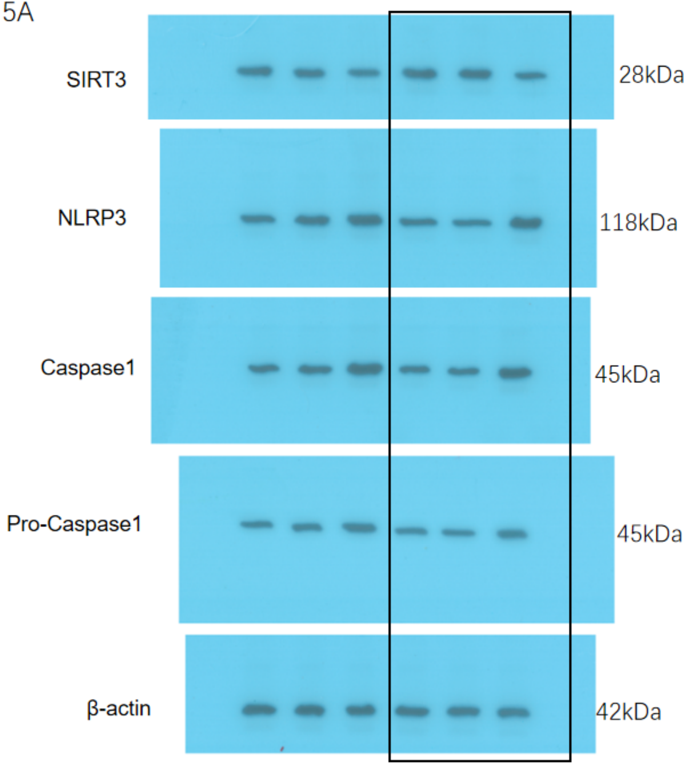

SIRT3
28kDa
NLRP3
118kDa
Caspase1
45kDa
Pro-Caspase1
45kDa
β-actin
42kDa

Fig 7A

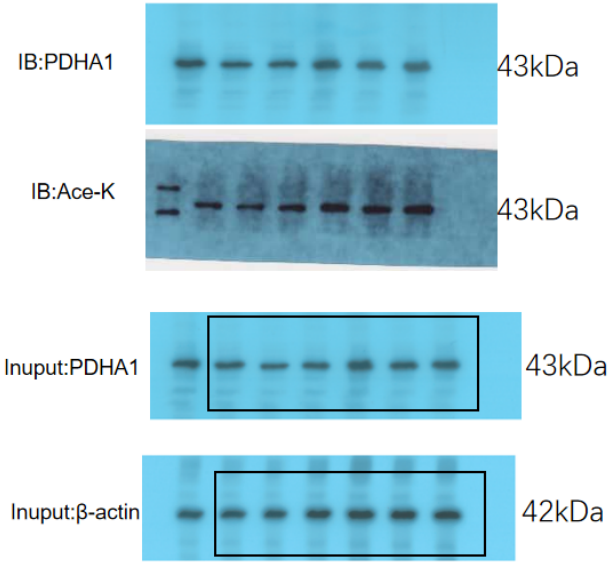

IB:PDHA1
43kDa
IB:Ace-K
43kDa
Inuput:PDHA1
43kDa
Inuput:β-actin
42kDa

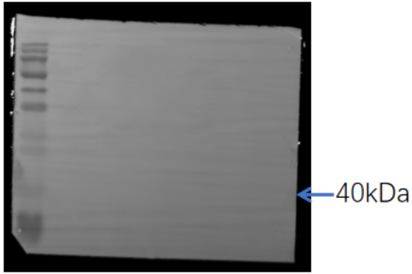

40kDa

Fig 7B

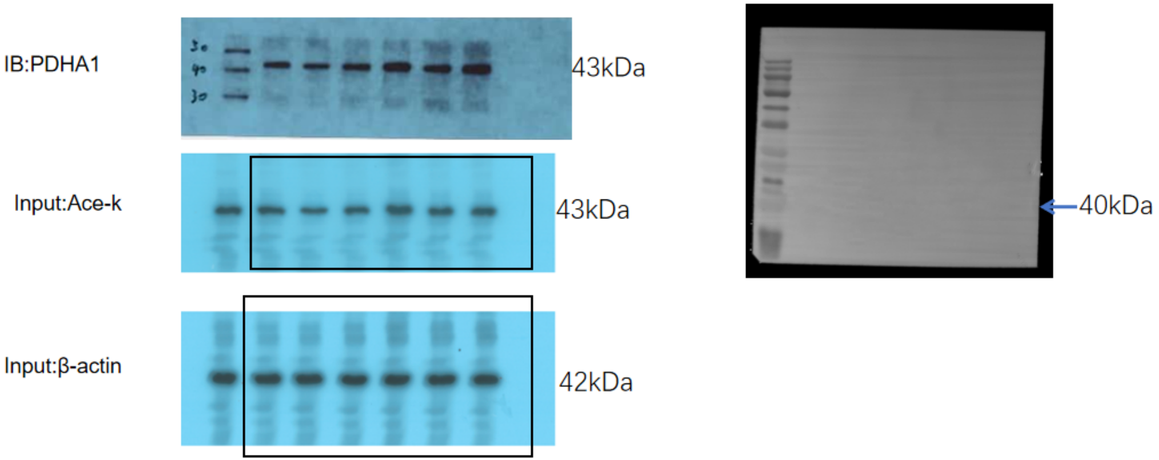

Fig 7C

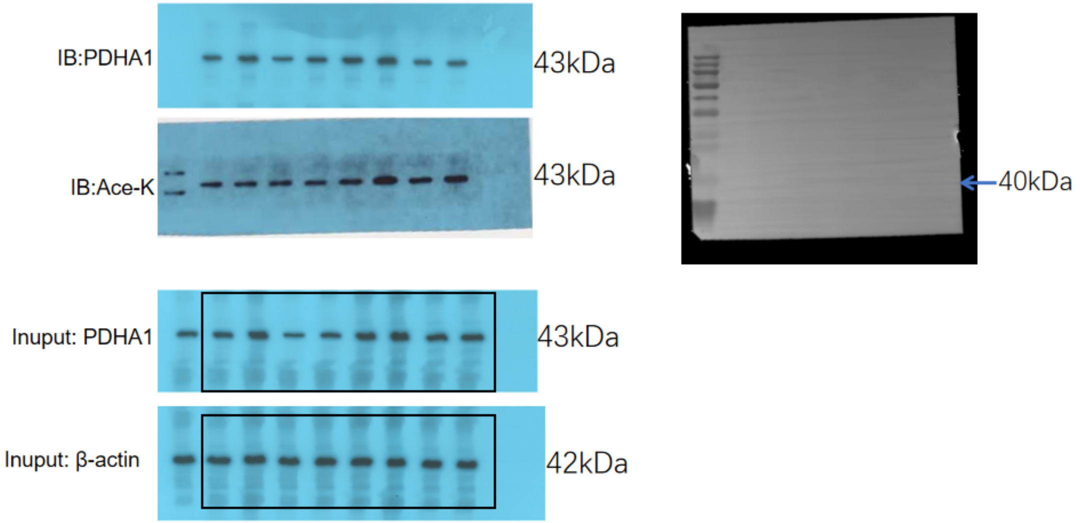
IB:PDHA1
43kDa
IB:Ace-K
43kDa
Inuput: PDHA1
43kDa
Inuput: β-actin
42kDa
40kDa

Fig 7D

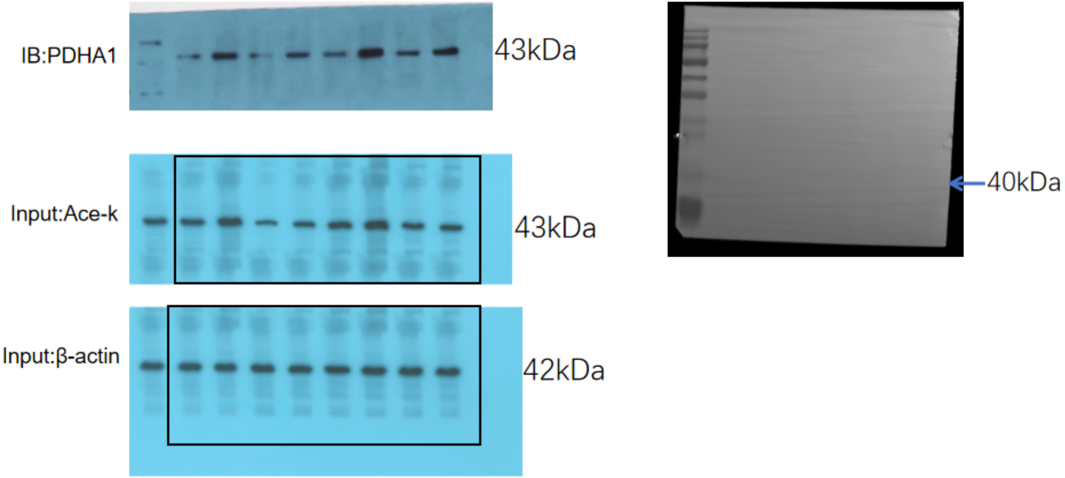

Supplement: Document S1. Data S1 [file mmc1.pdf]
